# Production and Commercialization of Traditional Agri-Food Products (TAPs) of Plant Origin in Campania: A Territorial-Scale Analysis

**DOI:** 10.3390/plants15213471

**Published:** 2026-11-06

**Authors:** Alessandro Borrelli, Farzaneh Zamani, Marco Esposito, Roberta Paradiso

**Affiliations:** 1 Department of Agricultural Sciences, University of Naples Federico II, 80143 Naples, Italy; alessandro.borrelli2@unina.it (A.B.); farzaneh.zamani@unina.it (F.Z.); 2 Prevention and Veterinary Public Health Unit, Directorate General for Health Protection and Coordination of the Regional Healthcare System, Campania Region, 80143 Naples, Italy; marco.esposito@regione.campania.it

**Keywords:** cultural heritage, organic farming, short supply chains, small-scale farming, local varieties, plant genetic resources, conservation strategies, agrobiodiversity

## Abstract

According to the list of Traditional Agri-food Products (TAPs) of the Italian Ministry of Agriculture, Food Sovereignty and Forestry, 1636 plant-based TAPs are currently registered in Italy. This study aimed at identifying strengths and constraints associated with the production, commercialization, and conservation status of plant-based TAPs selected in Campania. Data on all 246 TAPs recorded in the region were collected and analysed to assess their distribution in the 5 provinces. The conservation status was assessed by cross-referencing the TAPs list with the Regional Register of Plant Genetic Resources at Risk of Extinction and the National Register of Conservation Varieties. The study was conducted on a sample of 13 products, selected by edible organ (leaf, fruit, inflorescence, seed, and tuber/rhizome). Questionnaires concerning production and commercialization were distributed to two farmers per product. Most farms showed limited surfaces (<1 ha in 69.2%), with low yearly production levels (<10 q in 27% and 10–50 q in 38%). Cultivation mainly consists of organic farming (58%), reflecting the widespread adoption of low-input and environmentally sustainable practices, but entails higher production costs and operational constraints, affecting farm economic sustainability and competitiveness in broader markets. All farms distribute products at the local level, while 15% and 23% also reach national and European markets, respectively. Our results highlight the potential role of TAPs in supporting the sustainable utilisation of local plant genetic resources, territorial identity and rural food tourism. Strengthening the economic viability of plant-based TAPs may contribute to reducing the risk of genetic erosion and preserving Campania’s plant biodiversity and cultural heritage.

## 1. Introduction

The spread of Large-Scale Retail Distribution (LSRC) produced relevant changes in horticultural supply chains and in the consumption of plant products [1]. The expansion of LSRC chains and the implementation of logistics and trade strategies geared toward standardization promoted the continuous and extensive availability of fruits and vegetables, decreasing distribution costs and improving accessibility for a larger proportion of the population [2]. The economic relevance of large-scale retail distribution is reflected in the market: in 2018 in Italy, the three largest retail chains, *Coop*, *Conad* and *Selex*, collectively accounted for 34% of the national food retail market, compared with 61% held in Germany and the United Kingdom [2]. This highlights the significant role of major retail operators in food distribution and the widespread adoption of standardised commercial practices, despite differences between countries.

However, this approach implies varietal selection, standardization of supply, and policies promoting products with extended shelf-life, with negative consequences for biodiversity, local production systems, and the economy of small farms [3,4]. Particularly, Marín et al. [4] reported higher intraspecific diversity in farmers’ markets than in supermarkets. From an environmental perspective, comparing large-scale retail distribution and alternative food networks in Italy, a rate of 0.10 kg CO_2_-equivalent lower transport-related emissions per kg of oranges has been estimated for the alternative distribution system [5]. However, it is worth noting that the relative environmental performance depended on the product considered and logistical organization.

In this regard, alternative and local food systems (ALFSs) showed an emerging role in assuring the strength and sustainability of agri-food supply chains, notably during the COVID-19 pandemic [6]. Cross-country studies highlighted how ALFSs could quickly adapt to social and technological changes by fostering a greater participation among consumers and retailers in sustainable food approaches, while enhancing the production and consumption of traditional agri-food products. Accordingly, ALFSs represent a strategic approach to promote healthier diets, preserve agricultural biodiversity, and strengthen local economies, thereby reducing certain risks related to the LSRC model.

In this scenario, one of the novel initiatives receiving increasing attention is the development and diffusion of Short Food Supply Chains (SFSCs), together with other channels such as local markets, agricultural cooperatives, on-farm sales, and community-based platforms. These can promote a closer relationship between producers and consumers and can also support the marketing of products with unique features [7]. Recent studies also highlight the role of local labels and certification schemes in increasing consumer awareness and the commercialization of these products. At the same time, the literature points to several restrictions including producers’ limited access to distribution channels, low production capacity, and restricted marketing skills [8,9].

From this point of view, traditional agri-food products (TAPs) represent an essential part of Campania’s agricultural, cultural, and economic heritage [10]. Owing to the fertility of volcanic soils, climatic variability, and a deeply rooted rural tradition, many typical agricultural crops, including vegetables, legumes, fruits, and cereals, have evolved over the centuries, still comprising a defining feature of Campanian agri-food identity [11]. Beyond their fertility, volcanic soils can be characterized by distinctive water-retention properties, which may influence water availability to crops [12]. The high capacity of these soils to retain and release water may contribute to regulating moisture conditions during plant growth, potentially affecting physiological processes involved in the development of plant tissues. Furthermore, experimental evidence indicates that relative humidity affects the mechanical properties of lignocellulosic polymers [13], demonstrating the sensitivity of these materials to moisture conditions. Although these findings do not directly establish a relationship between soil moisture and lignocellulosic properties in growing plants, they provide a rationale to further investigate whether variations in water availability in volcanic soils may be associated with changes in anatomical traits of cultivated plants. Understanding these interactions could contribute to a more comprehensive assessment of the influence of volcanic soil on crop performance. These products result from agricultural practices handed down over time and are tightly related to the territory, playing a crucial role in safeguarding biodiversity. Recent studies highlight how traditional products are an important tool to ensure agrobiodiversity protection and regional rural heritage [14].

Beyond their cultural and economic importance, plant-based TAPs may contribute to the conservation of agrobiodiversity through the continued cultivation of local plant genetic resources, including traditional varieties and landraces [15]. However, the abandonment of traditional crops and the replacement of locally adapted varieties with modern cultivars may contribute to genetic erosion and the loss of crop diversity [16]. In this context, on-farm conservation represents an important strategy for maintaining plant genetic resources within their traditional agricultural environments, while supporting their sustainable utilisation and preserving local agricultural knowledge [15].

Plant TAPs have a crucial function in enhancing local economies and preserving traditional agricultural techniques. The existence of small farms and local supply chains allows for safeguarding sustainable production methods and supporting the connection between product and territory. Consistently, according to several studies on the Campanian agri-food system, the protection of traditional products is a key factor for territorial competitiveness and sustainability [17,18]. In addition, the integration of TAPs into touristic activities contributes to the resilient development of rural communities by supporting the local economy and strengthening the connection between travellers and territories, through an increasing attention to the distinctive features and geographical origin of local products [19,20]. Within these experiences, typical food becomes a tool for sharing the cultural heritage of local communities [21].

Specifically, the expression “Traditional Agri-food Products” denotes those products whose processing, preservation, and ripening techniques have been reinforced over time [22]. In addition, the term refers to products obtained using traditional production methods, over a period of no less than 25 years [23]. TAPs are categorized into 10 groups: non-alcoholic beverages, spirits, and liquors; cheeses; condiments; fats (butter, margarine, oils); fishes, mollusks, and crustacean products and their specific aquaculture techniques; fresh meats (and offal) and their preparations; fresh pasta and bakery products, including biscuits, confectionery, and pastries; plant products, processed or unprocessed; products of animal origin; and specialty food products.

This study aimed to characterize plant-based TAPs of the Campania region by distinguishing between products marketed in their natural state and processed products. The TAPs marketed in their natural state were further classified according to plant type into herbaceous or tree species, with herbaceous species additionally categorized based on the edible plant organ. Subsequently, 13 plant-based TAPs from Campania were investigated to examine their production and commercialization characteristics, considering different farms.

## 2. Results and Discussion

### 2.1. Identification and Classification of All Plant-Based TAPs of the Campania Region

#### Classification

**Figure 1:**
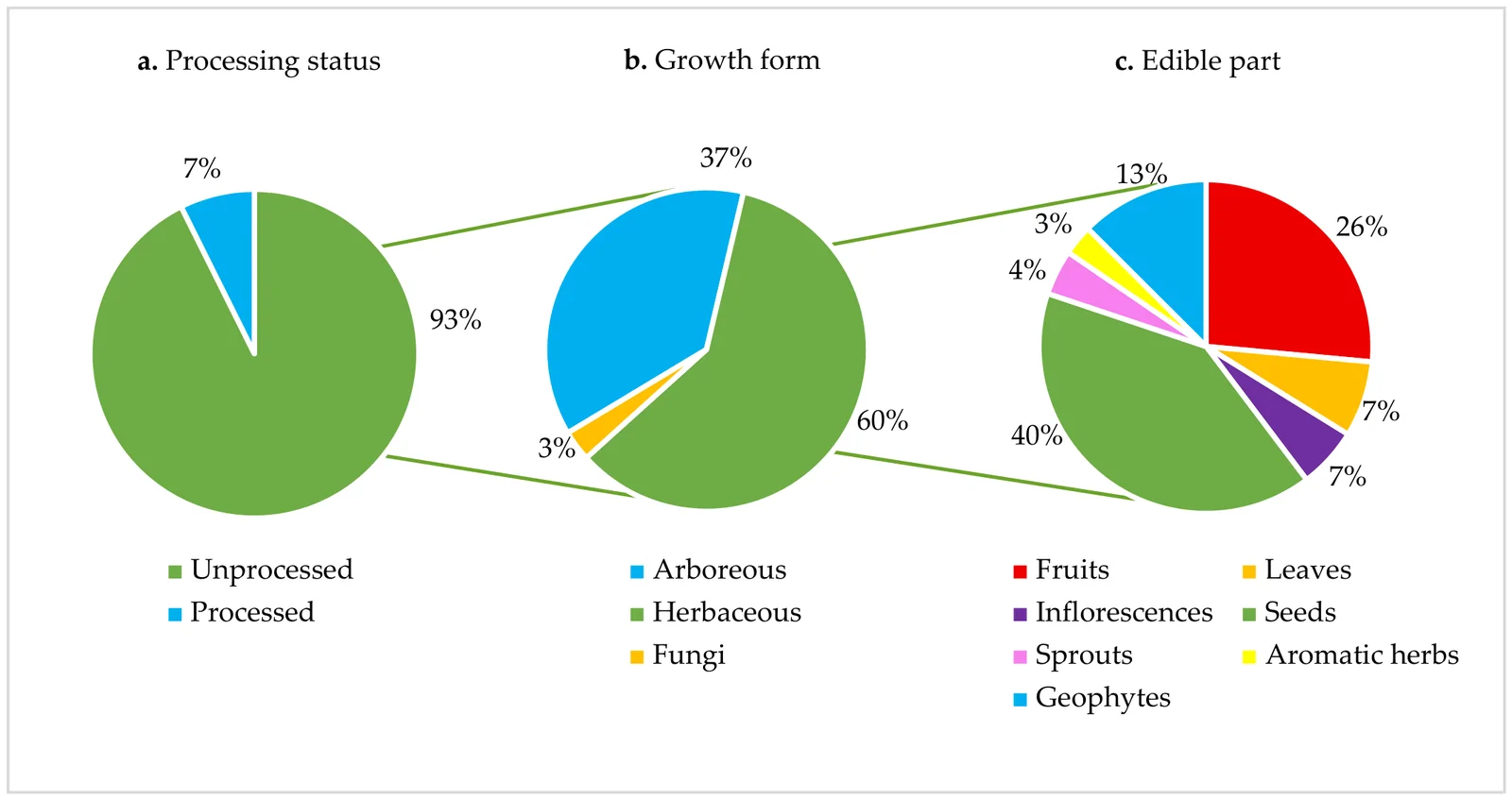
Classification of plant-based TAPs of Campania by processing status (**a**), growth form (**b**), and edible plant part (**c**), based on data from the 26th revision of the TAPs list (MASAF Ministerial Decree, 6 February 2026).

The dataset of plant products consisted of 93% unprocessed plant products and 7% processed products (Figure 1a). The predominance of unprocessed products reflects the close relationship between traditional food heritage and the use of minimally processed plant-based resources [14]. Unprocessed plant products were further classified according to plant type, with 60% of the products deriving from herbaceous species, 37% from arboreal species, and only 3% from fungi (Figure 1b). Focusing on herbaceous species, the most represented category consisted of seed-based products, which accounted for 40% of the total, followed by fruits (26%), hypogeous organs of geophytes (13%), leaves (7%), inflorescences (7%), sprouts (4%), and aromatic herbs (3%) (Figure 1c).

- Year of recognition and production classification

The systematic review of all official TAPs lists from 2000 to 2026 reveals a clear and consistent increase in the number of TAPs recorded in Campania, particularly in plant-based products. These products showed different production statuses across the region.

**Figure 2:**
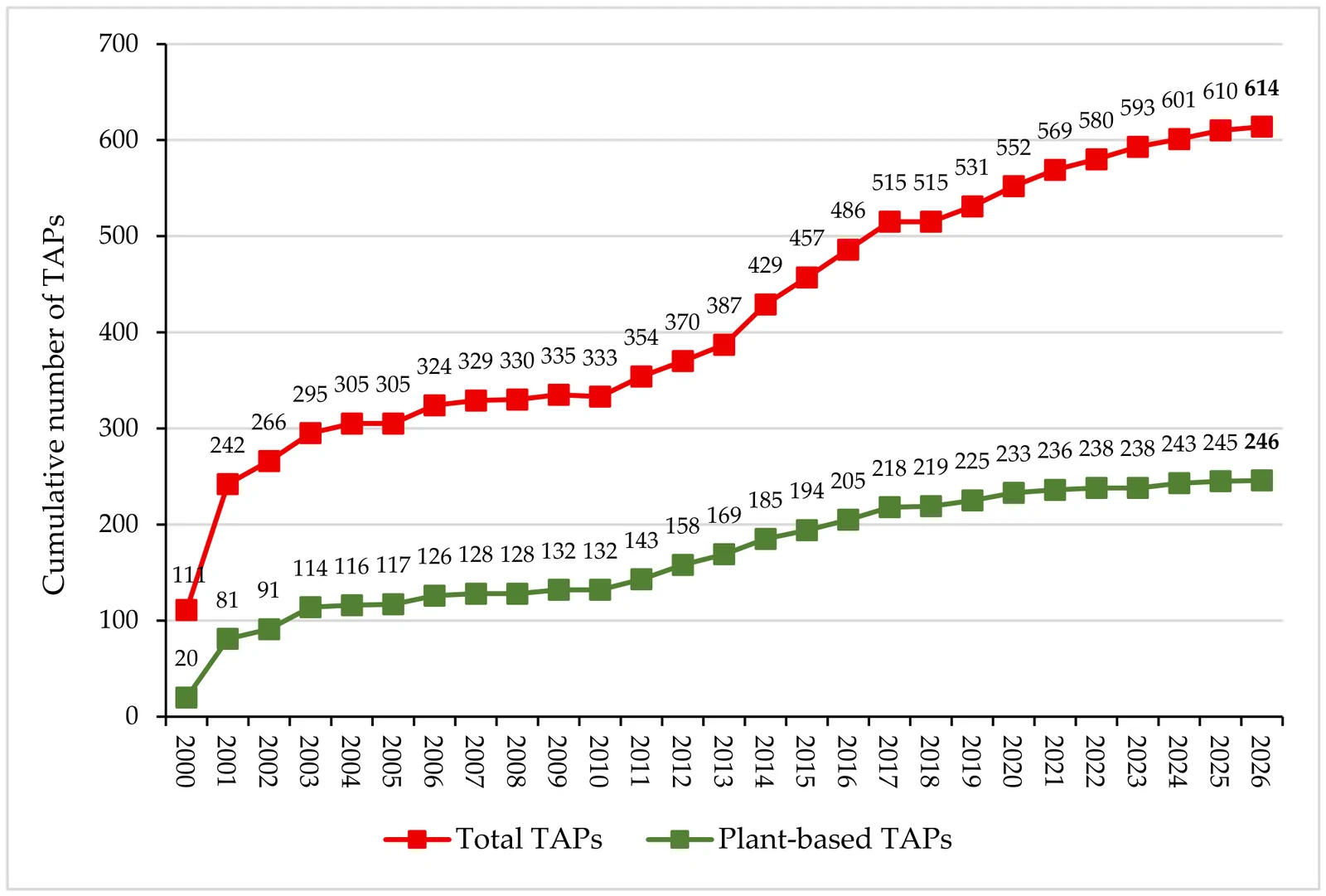
Temporal distribution of total TAPs and plant-based TAPs in the Campania region, according to the year of recognition.

The total number of TAPs in Campania rose from 111 in 2000 to 614 in 2026, a more than 5-fold increase (Figure 2) [24,25]. This trend demonstrates a progressive enrichment of the regional food resources, driven by enhanced identification processes, greater attention to rural biodiversity, and increasing appreciation of cultural and culinary heritage [26,27].

A similar pattern was observed in this Italian region for plant-based TAPs, which showed a considerable increase from 20 products recorded in 2000 to 246 in 2026. This growth reflects the increasing attention to the valorisation of local products as a relevant component of the regional agri-food traditions [14] and suggests a greater scientific and institutional attention to the preservation of local heritage [28]. Overall, these chronological trends show a gradual expansion of the TAP framework in Campania, both in total recognized products and within the plant-based category, highlighting the evolving recognition of traditional agri-food heritage. However, formal recognition alone may not be sufficient to ensure the long-term preservation and valorisation of these products. In Campania, agricultural districts provide a territorial framework through which local production can relate to broader rural development strategies [29]. Quality Agri-food Districts (DAQs) may facilitate cooperation among farmers, institutions, and other territorial actors, fostering knowledge exchange, innovation, and collective strategies for product valorisation. This perspective is consistent with the increasing emphasis of agricultural and rural development policies on multi-actor cooperation and innovation, particularly within the Agricultural Knowledge and Innovation System (AKIS) framework [30]. District-based networks may therefore represent a relevant opportunity for Campania’s plant-based TAPs, particularly for small-scale producers, by supporting product valorisation and value-chain integration while maintaining the strong territorial identity of traditional productions.

**Figure 3:**
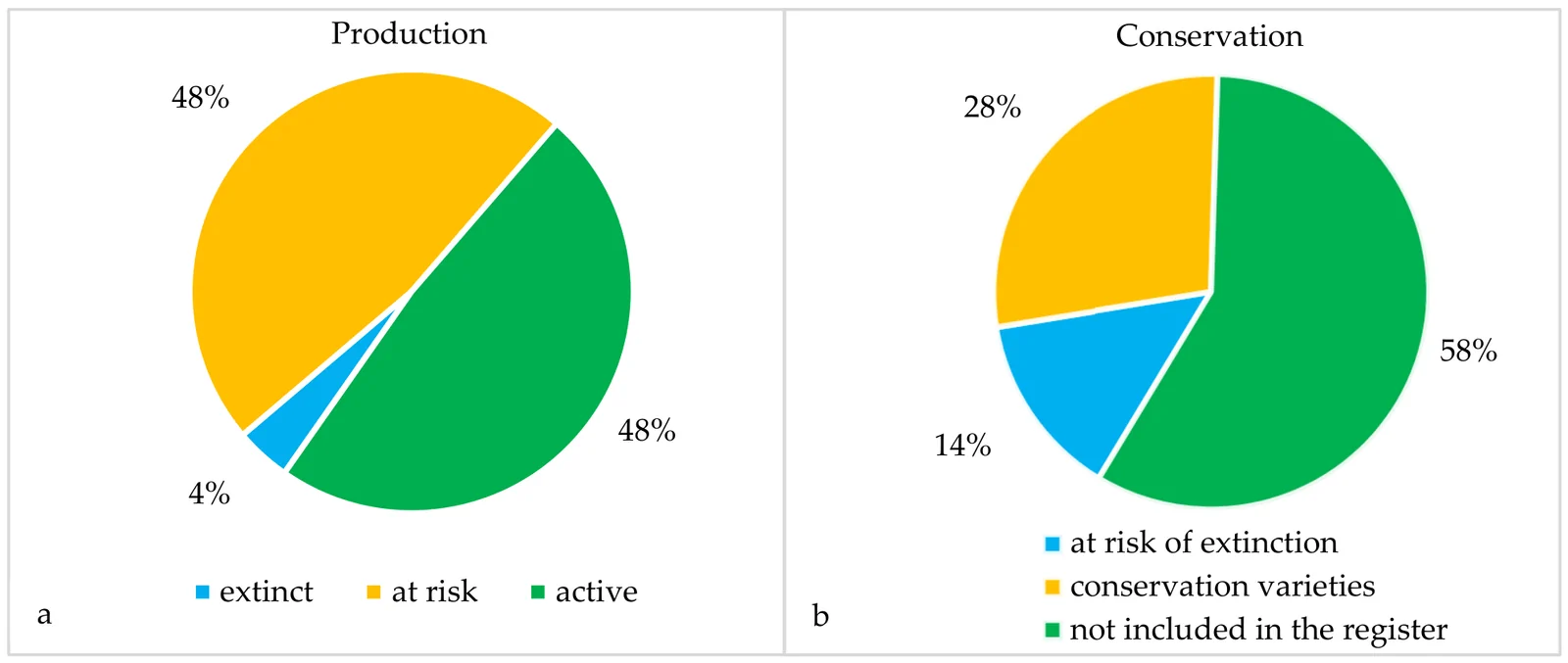
Production (**a**) and conservation status (**b**) of plant genetic resources associated with Campania’s plant-based TAPs. Production status was assessed using institutional documents published by the Campania Regional Department of Agriculture, while conservation grade was determined by cross-referencing the plant genetic resources associated with each TAP against the Regional Register of Plant Genetic Resources at Risk of Extinction and the National Register of Conservation Varieties.

In addition, the conservation and production status of plant TAPs shows that the products are distributed across three different categories: active, vulnerable, and extinct (Figure 3a). Almost half of the plant-based products (48%) are currently classified as active, indicating that they are still regularly produced and commercialized. A similar portion (48%) falls into vulnerable status, highlighting the need for targeted conservation and valorisation strategies to safeguard these traditional products and their associated agricultural heritage. Previous studies have identified limited production capacity, restricted market access and the erosion of traditional knowledge as potential threats to the continuity of traditional agri-food production [8]. Although these factors were not directly assessed in the present study, they provide a broader context for understanding the challenges faced by TAPs. The efforts of conservation should therefore consider not only the products themselves but also the underlying farming practices and local knowledge. A small fraction (4%) of plant TAPs is classified as extinct, indicating that their production has stopped or that they are no longer reported in current traditional practices. The occurrence of extinct products, together with the high proportion of vulnerable ones, highlights the ongoing risk of erosion of agrobiodiversity and landrace heritage [31].

Generally, this distribution describes a system in which a relevant part of local production may be recoverable, while also emphasizing the fragility of those currently at risk and the need for targeted conservation strategies to prevent further loss.

The cross-referencing of plant-based TAPs included in the National List with the Regional Register of Plant Genetic Resources at Risk of Extinction revealed that 42% of TAPs were associated with plant genotypes subjected to formal conservation frameworks (Figure 3b). Specifically, 14% were at risk of extinction and 28% were associated with conservation varieties, while the remaining 58% were not included in any of these categories. This finding highlights an important effort in the conservation of local traditional plant products. Indeed, the observed association between 42% of the analysed TAPs and plant genetic resources included in the Regional Register suggests that maintaining these traditional productions may contribute to the on-farm conservation of adapted local genetic resources, as also supported by previous studies on the conservation of traditional crop varieties [15]. Their inclusion in the Regional Register provides a formal framework for conservation actions aimed at preventing the genetic erosion and loss of locally adapted plant genotypes. In Campania, these resources are supported through an integrated conservation system involving in situ*/on-farm* conservation by custodian farmers and ex situ conservation in regional germplasm banks.

### 2.2. Selected Plant-Based TAPs

The analysis of 13 TAPs shows small-scale production, predominantly local markets, widespread sustainable practices, and limited production capacity. This results in low scalability and a restricted presence in national and European markets (Table 1). These characteristics highlight the potential relevance of regional policy instruments, such as Agri-food Quality Districts (DAQs) and the Campania Rural Development Complement 2023–2027, in addressing the challenges faced by small-scale TAP producers. These frameworks could promote cooperation, improve market access, and support product valorisation while preserving traditional production systems and local agrobiodiversity.

- Production status and other recognitions of selected TAPs

**Figure 4:**
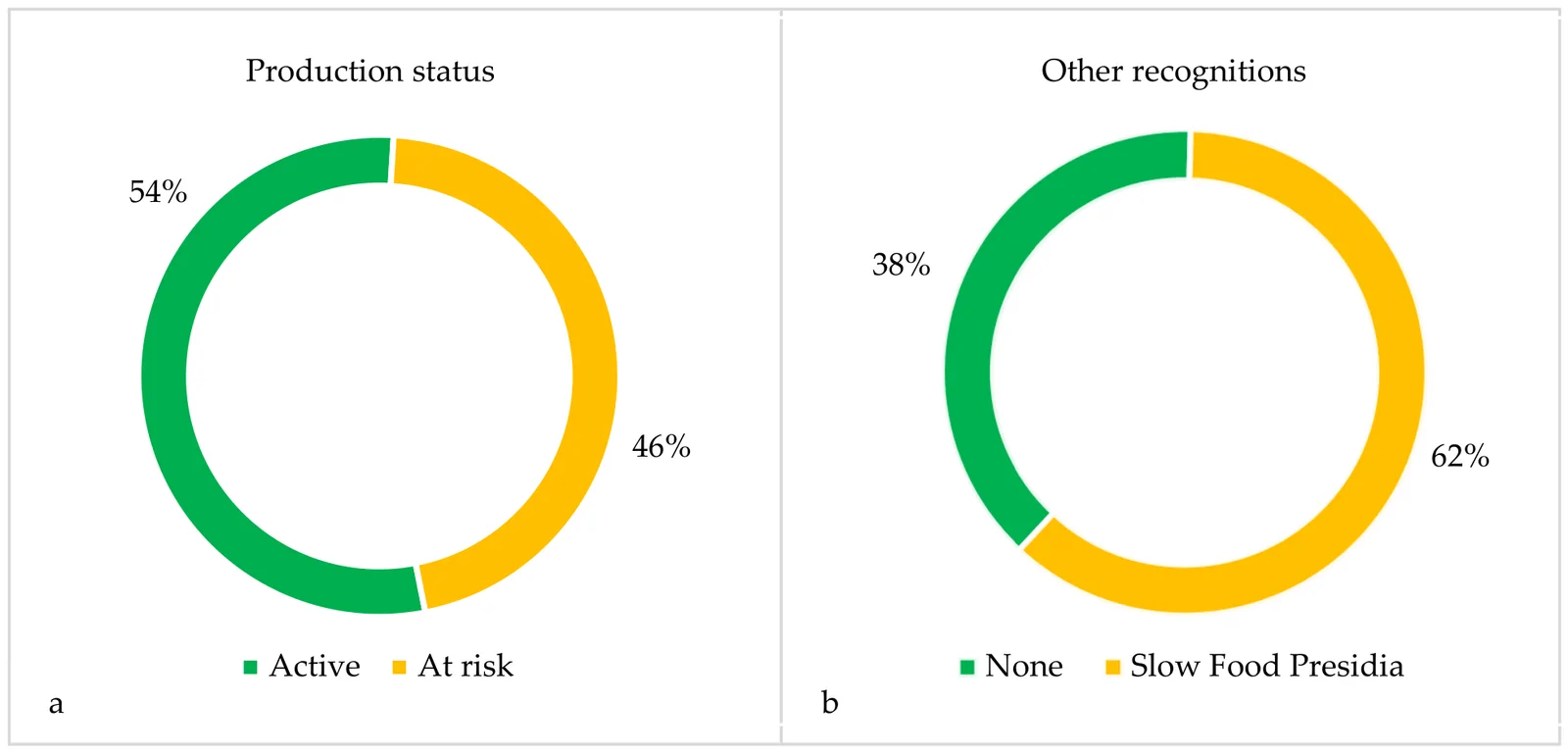
Production status (**a**) and additional recognitions (**b**) of 13 selected plant-based TAPs in 2026, according to survey data collected from 26 farms in Campania.

The production status of the 13 investigated products reveals that 54% are presently classified as regularly produced and 46% are considered at risk of extinction (Table 1; Figure 4a). Statistical analysis revealed significant differences in annual production and cultivated area between the two production status categories (Table 2). Specifically, actively produced TAPs showed significantly higher annual production (1774.6 q) and larger cultivated areas (6.8 ha) than those classified as at risk of extinction (23.8 q and 0.44 ha, respectively). This distribution reflects trends occurring in small-scale and traditional agri-food systems, where limited economic viability and generational turnover negatively affect the continuity of farming activities [27].

Strambi (2010) [32] highlighted that the intergenerational transmission of production methods and knowledge within local communities, closely related to their territories of origin, can support the preservation of traditional products. Many TAPs were often characterized by a critical conservation status or limited production levels, placing them in a particularly vulnerable state of abandonment, due to agricultural modernization processes and the widespread adoption of more productive commercial varieties.

**Figure 5:**
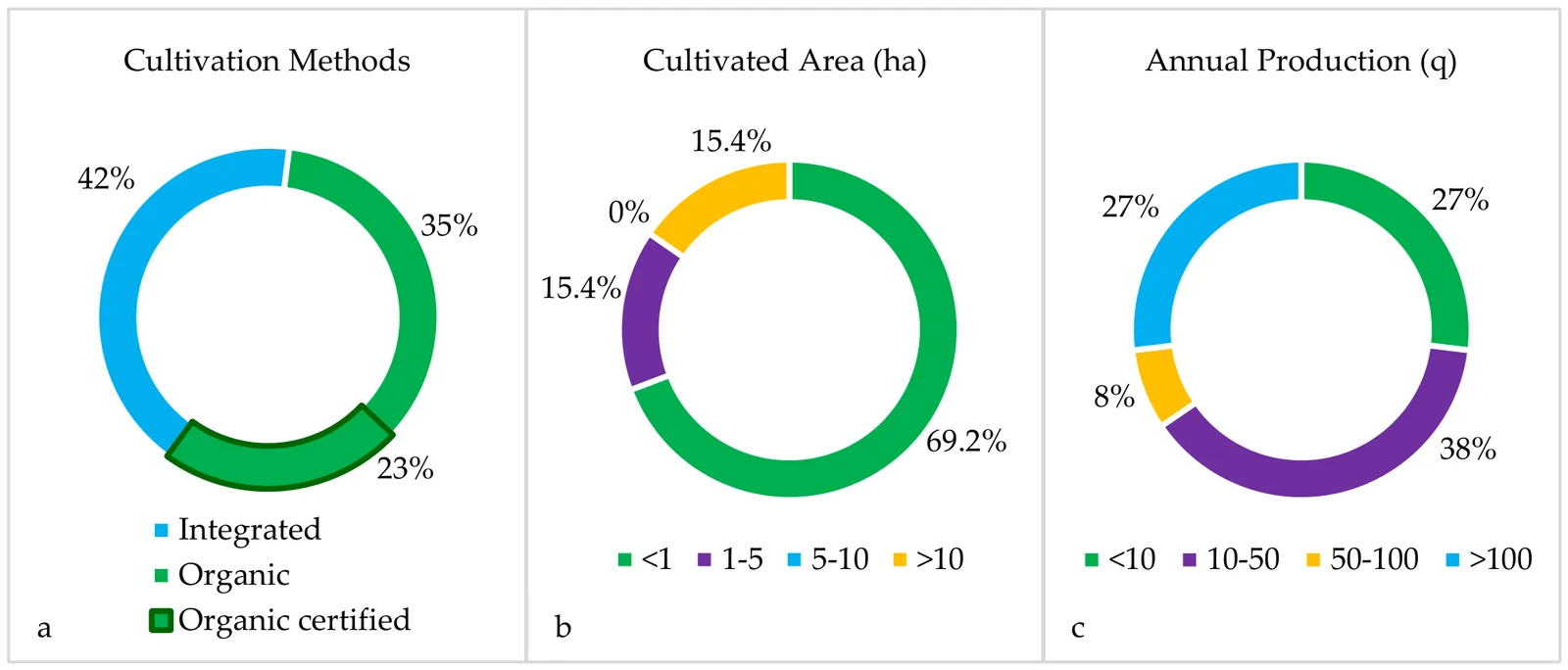
Agronomic practices (**a**) cultivated area (**b**), and annual production (**c**) of 13 selected plant-based TAPs in 2026, based on survey data from 26 farms in Campania.

Considering other labels, 38% of the companies present no additional certifications or recognition, while 62% are part of the Slow Food Presidia network, and 23% hold certified organic status (Figure 4b and Figure 5a). The prominence of Slow Food participation emphasizes the crucial role of labels in local food heritage [33,34].

- Cultivation practices, production capacity, and market distribution of selected TAPs

In terms of cultivation practices, 42% of the selected farms adopted integrated farming systems, while 58% declared organic management, though only 23% of them presented organic certification (Figure 5a). Despite the higher proportion of farms adopting organic practices, significant differences were observed between cultivation systems in terms of annual production and cultivated area (Table 2). Farms adopting integrated farming systems exhibited significantly higher annual production (2256.5 q) than organically managed farms (20.6 q). Similarly, the mean cultivated area was significantly larger under integrated farming (8.3 ha) than under organic management (0.6 ha). This moderate frequency of organic practices is in line with the growing transition toward low-impact agricultural systems observed in Mediterranean agroecosystems [35]. In addition, the coexistence of integrated and organic systems points to a different uptake of sustainability standards among producers.

TAPs are strongly related to local traditions and rural identity and may be characterized by limited cultivation areas or reduced commercial availability [26]. Moreover, the progressive abandonment of traditional crop varieties may contribute to genetic erosion [36], highlighting the importance of their continued cultivation and conservation. In this context, safeguarding traditional crop varieties requires an integrated approach combining the conservation of plant genetic resources with the continuity of local farming practices. Supporting cooperation among small-scale producers, facilitating the transmission of traditional agricultural knowledge and improving access to niche markets may contribute to the long-term viability of traditional agri-food production systems while preserving their distinctive characteristics [8]. Such measures could also strengthen the role of TAPs in maintaining agrobiodiversity and preserving the cultural heritage of rural communities. For 69.2% of the selected farms, the cultivated area allocated to TAP is smaller than 1 hectare, confirming a strong smallholder-based production structure (Figure 5b). This segmentation is widely recognised as a constraining factor for scaling production and market competitiveness [37,38], and is consistent with the low annual production observed and highlights the vulnerability of this production system. However, although small surfaces limit the production volumes and market access, they should not necessarily be interpreted as a structural weakness only. Small-scale TAP cultivation may also contribute to the resilience of local agriculture while supporting the continuity of traditional practices, crop diversity, and territorial identity.

Particularly, 38% of farms show annual production between 10 and 50 quintals, while 27% present less than 10 quintals and more than 100 quintals, respectively. Only 8% of farmers achieve production levels between 50 and 100 quintals (Figure 5c). This mixed distribution suggests a deficit of mid-scale production systems, which is often evaluated as a crucial element for regulating supply chains and improving market access [38]. The limited portion of intermediate production levels may partly reflect structural and economic constraints that hinder the transition from small-scale to medium-scale farms. Land fragmentation, limited investment capacity and difficulty in achieving economies of scale may restrict opportunities for farm expansion [38]. Furthermore, demographic factors, particularly the ageing of farm managers and limited generational succession, may limit opportunities for farm growth [39]. Together, these factors could contribute to the observed distribution of production volumes. However, their specific influence was not directly assessed in the present study.

**Figure 6:**
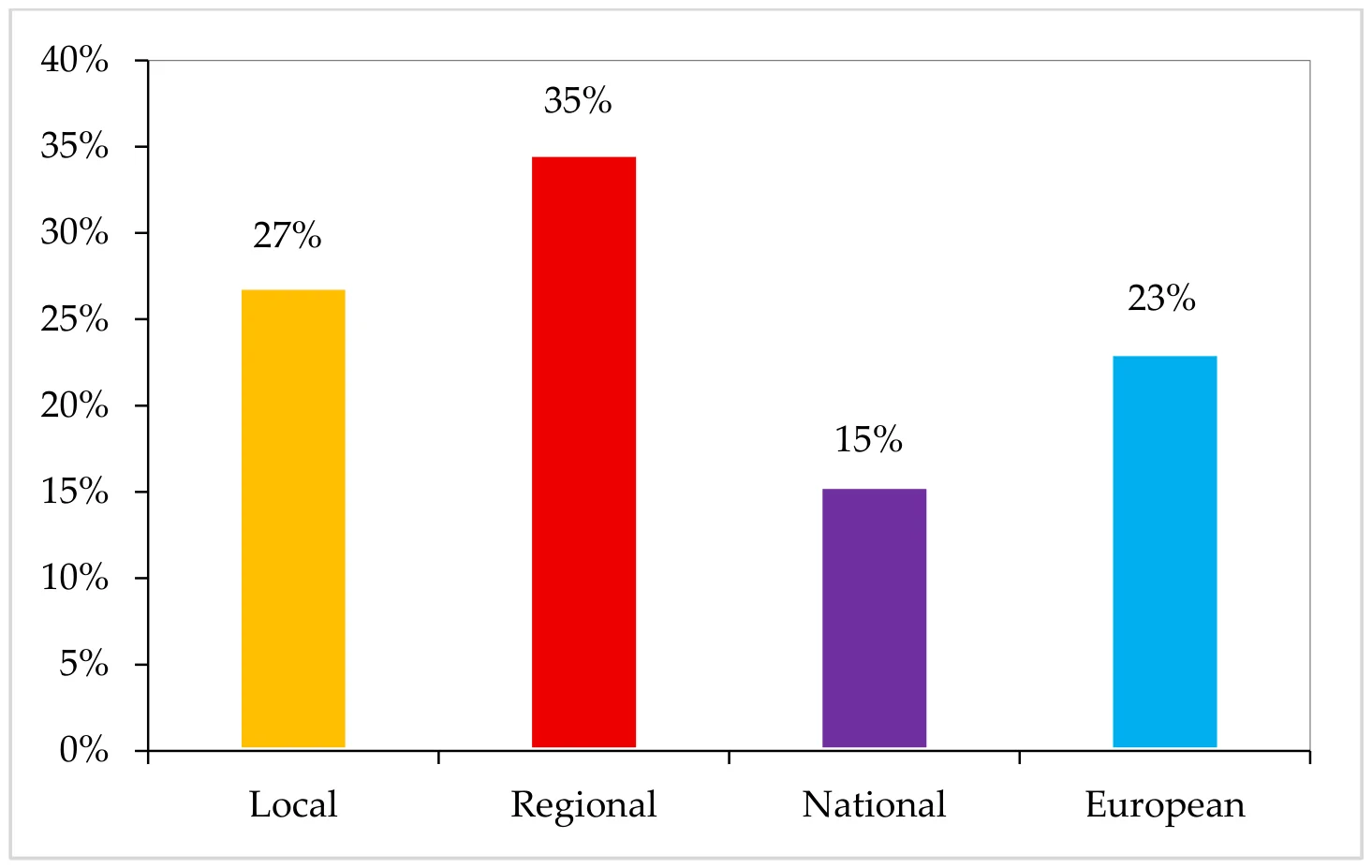
Market distribution levels of 13 selected plant-based TAPs in 2026, according to survey data collected from 26 farms in Campania.

The constrained production capacity, in combination with inadequate marketing strategies, limits these products primarily to niche or local markets (Figure 6). Indeed, 27% of selected farms operate only at the local market, and 35% also reach regional markets. Participation decreases progressively as geographical scale expands; only 15% reach national markets, while just 23% reach the European market. These differences in market distribution were further supported by the statistical analysis, which revealed significant differences among market categories for both annual production and cultivated area (Table 2). Farms operating in European markets exhibited the highest mean annual production (3924.2 q) and cultivated area (13.9 ha), whereas those operating in local markets showed considerably lower values (66.6 q and 0.3 ha, respectively). Tukey’s post-hoc test indicated that farms operating in European markets differed significantly from those operating in local and regional markets for both variables, while farms operating in national markets showed intermediate values without significant differences from the other market categories.

Regarding geographical distribution, no statistically significant differences were detected among the five provinces of Campania in either annual production or cultivated area (Table 2), despite the numerical variability observed across provinces.

Overall, this evidence suggests that the predominance of small-scale farms in Campania limits the exploitation of economies of scale and constrains productivity growth, thereby reducing the capacity of farms to compete effectively in wider national and international markets [19].

- Strategies for TAP valorisation

The institutional and policy opportunities outlined above are particularly relevant to the 13 selected TAPs, as their preservation and commercial development may benefit from coordinated territorial strategies.

For producers operating on a limited scale, collective initiatives represent an opportunity to improve visibility and market access while preserving the strong territorial identity characterising traditional agri-food products. In this regard, Agri-food Quality Districts (DAQs) could provide a useful setting for encouraging dialogue and cooperation among farmers, local institutions, and other stakeholders involved in rural development. Such collective approaches, together with measures supporting rural development, could help small producers overcome market marginalization through cooperation, shared resources, and product differentiation, without necessarily requiring an expansion of cultivated surfaces or a shift towards industrial-scale production [29].

At the same time, several instruments already available within the Campania Rural Development Complement 2023–2027 (CSR) offer opportunities that are relevant, albeit indirectly, to traditional products. Measures supporting innovation, cooperation, and quality enhancement could help producers address some of the challenges associated with small-scale production, particularly regarding market access and product recognition. Investments in traceability systems, digital communication, and supply-chain coordination may be especially important for increasing consumer awareness without weakening the connection between products and their territories of origin.

Promotion represents another key aspect. Despite their cultural and historical significance, many traditional products remain less known outside local contexts. Initiatives such as Promotion of Quality Products (SRG10), which supports collective communication and advertising activities, suggest the potential value of coordinated actions aimed at increasing the visibility of local products and their distinctive attributes to a wider audience [30].

## 3. Materials and Methods

The study was carried out in the Campania region, in the south of Italy, a territory characterised by great agrobiodiversity and a long tradition of typical plant products. The research focused on the 26th list of Traditional Agri-food Products (TAPs), officially recognized by the Italian Ministry of Agriculture, Food Sovereignty and Forestry—MASAF [25].

### 3.1. Characterization of All Plant-Based TAPs of the Campania Region

Data were processed using Microsoft Excel (Microsoft Corporation, Redmond, WA, USA) and the elaboration was based on regional institutional sources and all official TAPs lists issued by MASAF from 2000 to 2026, including the ministerial revisions published during the study period. Previous official lists were considered to assess the annual increase in the total number of TAPs in Campania and in those belonging to the category of “processed or unprocessed plant-based products” (Figure 2), while the 2026 official list was used as the reference for the characterization of the TAPs included in the study. For each TAP of Campania, information was collected considering the category of “processed or unprocessed plant-based products” and edible plant organs, such as fruit, leaf, bulb, tuber, seed, inflorescence, stem, and root.

In addition, a chronological analysis of TAP recognition was conducted by comparing recognized plant-based TAPs with the total number of TAPs registered in Campania from 2000 to 2026 to highlight trends in their institutional recognition and valorisation over time.

The production classification of plant-based TAPs was reviewed using institutional documents (Campania Regional Department of Agriculture), bibliographic sources, and on-site verification with local farmers. Products were classified as active, exposed to the risk of extinction, or extinct. These categories were explicitly indicated in the official product records held in the archives of the Campania Regional Department of Agriculture and were not established by the authors. These records were used together with bibliographic sources and on-site verification with local farmers to review the production status of the products.

Finally, to further assess the conservation status of plant-based TAPs, those included in the Italian National List were cross-referenced with the Campania Region’s Regional Register of Plant Genetic Resources at Risk of Extinction (*Repertorio regionale delle risorse genetiche vegetali a rischio di estinzione*) [40]. The register, established within the regional framework for the conservation of agricultural genetic resources, includes plant genotypes of regional interest considered at risk of extinction. It represents one of the main tools adopted by the Campania Region for safeguarding agricultural biodiversity. The comparison was performed by matching the plant genetic resource associated with each TAP with the resources officially included in the Regional Register.

### 3.2. Farm-Level Survey and Characterization of Selected Plant-Based TAPs of the Campania Region

**Figure 7:**
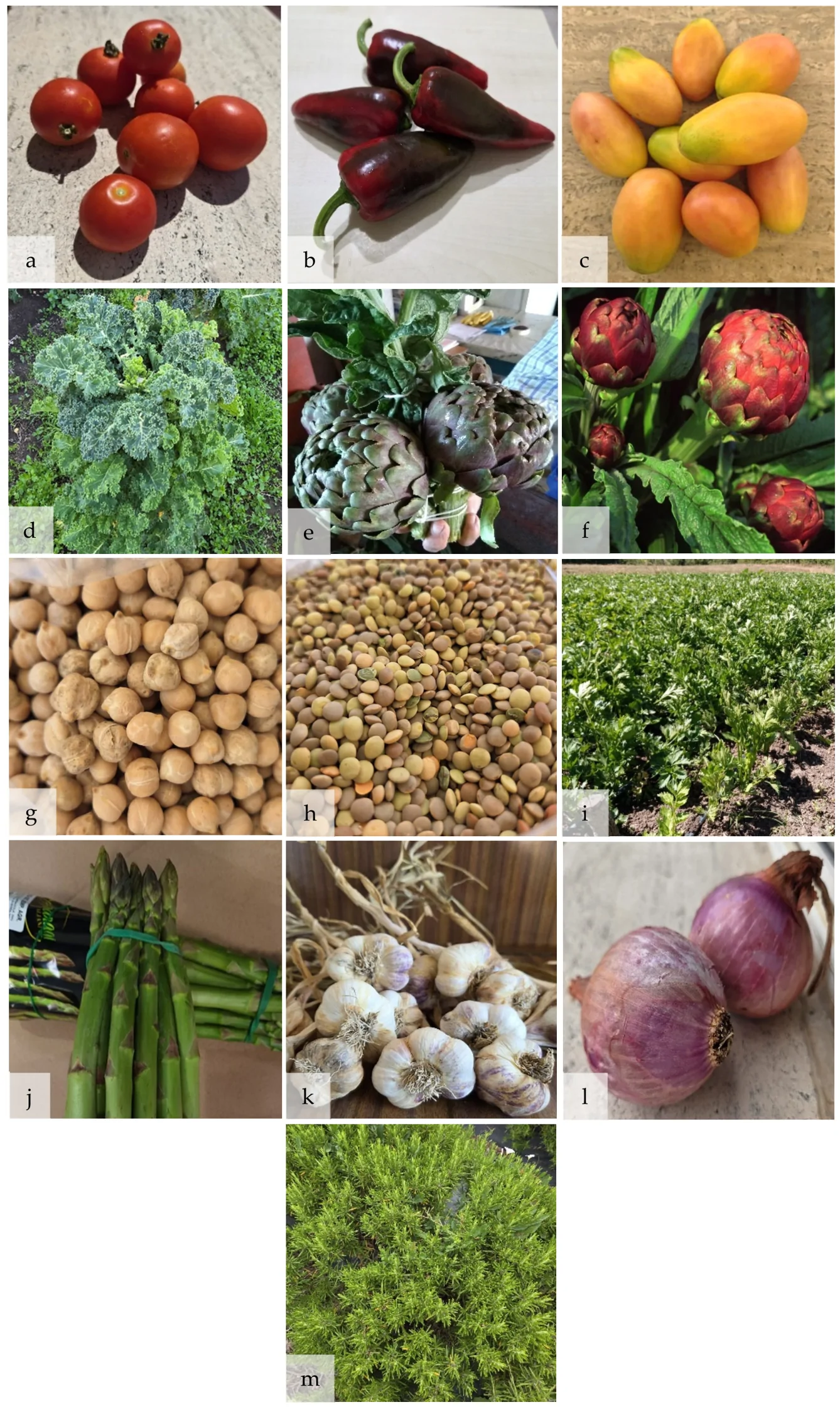
Plant-based TAPs selected for the study. (**a**)—*Solanum lycopersicum* L. ‘Seccagno di Gesualdo’; (**b**)—*Capsicum annuum* L. ‘Quarantino di San Salvatore’; (**c**)—*Solanum lycopersicum* L. ‘Rofrano’; (**d**)—*Brassica oleracea* L. var. acephala ‘Torzella’; (**e**)—*Cynara cardunculus* L. var. *scolymus* ‘Pietrelcina’; (**f**)—*Cynara cardunculus* L. var. *scolymus* ‘Castellammare’; (**g**)—*Cicer arietinum* L. ‘Cicerale’; (**h**)—*Lens culinaris* Medik ‘Valle Agricola’; (**i**)—*Apium graveolens* L. ‘Gesualdo’; (**j**)—*Asparagus officinalis* L. ‘Napoletano’; (**k**)—*Allium sativum* L. ‘Ufita’; (**l**)—*Allium cepa* L. ‘Alifana’; (**m**)—*Salvia rosmarinus* L. ‘Cilento’.

A sample of 13 plant products was selected from the TAPs list of the 26th revision of MASAF for the farm-level investigation (Figure 7). The selection process aimed to include distinct products in terms of geographical distribution, cultivation features, level of market diffusion, and conservation status. Specifically, a purposive sampling approach was adopted to ensure a balanced geographical distribution across the five provinces of Campania and to include different edible plant organs, according to the product classification adopted in this study. The sample size was determined considering the feasibility of the farm-level survey.

The selected TAPs included only unprocessed products from five provinces of Campania: Avellino, Benevento, Caserta, Napoli, and Salerno, thus representing the biodiversity, pedoclimatic conditions and farming contexts across the region.

For each selected product, based on the type of edible organ, two farms were selected based on the continuity of cultivation methods, the representativeness of local production systems, historical knowledge of the traditional product, and the willingness of farmers to participate in the study. Data collection was performed through a questionnaire, aimed at collecting information about cultivation practices and area, production characteristics, additional certification, and marketing channels.

The farmers were contacted personally, and each company finalized the questionnaire specifically related to its corresponding TAP. Overall, 26 questionnaires were collected, with two companies for each of the 13 selected products.

### 3.3. Data Processing

Data were organized into a specific database based on botanical classification, production status, cultivation method, cultivated surface, average annual production, and marketing channels.

Data were processed Microsoft Excel (Microsoft Corporation, Redmond, WA, USA) using a descriptive data analysis and presented through summary tables and graphical representations to describe the distribution of the selected TAPs across the Campania region and their main characteristics. Data were analyzed using Minitab 19 (version 19; Minitab, LLC, State College, PA, USA) and one-way analysis of variance (ANOVA) was performed to assess differences in annual production and cultivated area across groups defined by each of the following factors: market distribution, cultivation practices, production status, and geographical location. Prior to the analysis, the assumptions of normality of residuals and homogeneity of variances were assessed. Since the original data did not meet these assumptions, a base-10 logarithmic (log10) transformation was applied before performing ANOVA. When the F-test indicated statistically significant differences (*p* < 0.05), Tukey’s honestly significant difference (HSD) post-hoc test was used to identify significant pairwise differences among groups. Descriptive statistics were expressed as mean ± standard deviation (SD), and the significance threshold was set at *p* < 0.05.

## 4. Conclusions

Most of the selected farms are characterized by small, cultivated areas (<1 ha in 69.2%) and primarily operate in small-scale markets (local and regional for 62%). These features may limit their economic sustainability and commercial competitiveness in national and international markets. On the other hand, local farms acting in marginal contexts contribute to enhancing landscape diversity and preserving traditional methods. Accordingly, the leading trend towards local and regional markets confirms the distinctive territorial identity of Traditional Agrifood Products (TAPs) and highlights the importance of improving promotional activities and comprehensive marketing policies. Furthermore, the widespread application of integrated farming practices and long-established organic agriculture (both formally established for over 25 years) exhibits an increasing focus on sustainability. The expansion of marketing channels beyond local and regional commercial areas may offer opportunities to increase product visibility and strengthen the economic viability of plant-based TAPs.

Cultivation of plant-based TAPs associated with local plant genetic resources may contribute to the on-farm conservation and the sustainable utilisation of agrobiodiversity. Supporting traditional production systems and their economic viability could therefore help reduce the risk of genetic erosion while preserving Campania’s agricultural and cultural heritage. In this context, the Regional Register of Plant Genetic Resources at Risk of Extinction represents an important tool for safeguarding plant genetic resources. Furthermore, the involvement of institutions, producers, and consumers emerges as an essential condition to safeguard the preservation, valorisation, and transmission of plant-based TAPs.

The prevalence of small farms appears consistent with the historical and productive characteristics of the regional agricultural landscape. However, the concentration of commercial activities within local and regional markets raises questions about how the economic potential of plant-based TAPs could be further enhanced while preserving their strong connection to place and tradition.

In this context, institutional policies and initiatives, such as Agri-food Quality Districts (DAQs), Campania Rural Development Complement 2023–2027 (CSR), and Promotion of Quality Products (SRG10), represent valuable tools to sustain traditional products, protect agrobiodiversity and genetic resources, and help local economies.

More broadly, the results of this work highlight that the value of traditional agri-food products cannot be understood solely in economic terms. These products embody local knowledge, agricultural traditions, genetic resources, and landscapes with cultural value that have developed over time through the interaction between communities and their environment. In this sense, traditional products represent both an economic resource and an important component of the cultural and environmental heritage of rural areas.

The future activity of the research project will extend the survey to a larger number of farms and assess the economic sustainability and market opportunities of plant-based TAPs of the Campania region over time.

## Figures and Tables

**Table 1:** Overview of the main features of 13 selected plant-based TAPs from the Campania region.

Species	Local Variety	Product Category	Province of Production	Production Status	Year of Recognition
*Solanum lycopersicum* L.	‘Seccagno di Gesualdo’	Fruit	Avellino	at risk	2012
	‘Rofrano’	Fruit	Salerno	at risk	2012
*Capsicum annuum* L.	‘Quarantino di San Salvatore’	Fruit	Benevento	active	2012
*Brassica oleracea* L. var. *acephala*	‘Torzella’	Leaf	Napoli	at risk	2007
*Cynara cardunculus* L. var. *scolymus*	‘Castellammare’	Inflorescence	Napoli	at risk	2001
	‘Pietrelcina’	Inflorescence	Benevento	active	2000
*Lens culinaris* Medik	‘Valle agricola’	Seed	Caserta	at risk	2001
*Cicer arietinum* L.	‘Cicerale’	Seed	Salerno	active	2002
*Asparagus officinalis* L.	‘Napoletano’	Sprout	Napoli	active	2024
*Apium graveolens* L.	‘Gesualdo’	Sprout	Avellino	at risk	2017
*Salvia rosmarinus* L.	‘Cilento’	Aromatic herb	Salerno	active	2024
*Allium cepa* L.	‘Alifana’	Geophyte	Caserta	active	2013
*A. sativum* L.	‘Ufita’	Geophyte	Avellino	active	2001

**Table 2:** Characteristics of the Selected Traditional Agri-Food Products (TAPs).

Characteristics of the Selected Farms	Annual Production (q)	Cultivated Area (ha)
Market distribution		
Local	66.6 ± 91.3 b	0.3 ± 0.1 b
Regional	17.1 ± 14.2 b	0.6 ± 0.6 b
National	241.3 ± 373.0 ab	2.3 ± 2.0 ab
European	3924.2 ± 4534.0 a	13.9 ± 11.7 a
Cultivation practices		
Integrated farming	2256.5 ± 3740.3 a	8.3 ± 10.5 a
Organic farming	20.6 ± 15.9 b	0.6 ± 0.7 b
Production status		
Active	1774.6 ± 3417.4 a	6.8 ± 9.8 a
At risk	23.8 ± 19.8 b	0.42 ± 0.4 b
Production area		
Avellino	146.7 ± 230.1	1.1 ± 1.9
Benevento	109.7 ± 104.6	1.0 ± 1.0
Caserta	19.8 ± 26.9	0.8 ± 0.6
Napoli	947.8 ± 1399.9	6.6 ± 9.6
Salerno	3007.5 ± 5014.6	7.9 ± 12.2
Market distribution	**	**
Cultivation practices	***	**
Production status	*	**
Production area	ns	ns

ns, *, **, ***: Not significant or significant differences at *p* ≤ 0.05, 0.01, and 0.001, respectively. Different letters indicate significantly different values according to Tukey’s multiple comparison test (*p* ≤ 0.05).

## Data Availability

The literature sources of this review article will be made available by the authors upon request.

## Abbreviations

TAPs	Traditional agri-food products
LSRC	Large-scale retail distribution
